# Mitochondrial energetics with transmembrane electrostatically localized protons: do we have a thermotrophic feature?

**DOI:** 10.1038/s41598-021-93853-x

**Published:** 2021-07-16

**Authors:** James Weifu Lee

**Affiliations:** Department of Chemistry and Biochemistry, Old Dominion University, Norfolk, VA 23529 USA

**Keywords:** Bioenergetics, Biophysical chemistry

## Abstract

Transmembrane electrostatically localized protons (TELP) theory has been recently recognized as an important addition over the classic Mitchell’s chemiosmosis; thus, the proton motive force (pmf) is largely contributed from TELP near the membrane. As an extension to this theory, a novel phenomenon of mitochondrial thermotrophic function is now characterized by biophysical analyses of pmf in relation to the TELP concentrations at the liquid-membrane interface. This leads to the conclusion that the oxidative phosphorylation also utilizes environmental heat energy associated with the thermal kinetic energy (*k*_*B*_*T*) of TELP in mitochondria. The local pmf is now calculated to be in a range from 300 to 340 mV while the classic pmf (which underestimates the total pmf) is in a range from 60 to 210 mV in relation to a range of membrane potentials from 50 to 200 mV. Depending on TELP concentrations in mitochondria, this thermotrophic function raises pmf significantly by a factor of 2.6 to sixfold over the classic pmf. Therefore, mitochondria are capable of effectively utilizing the environmental heat energy with TELP for the synthesis of ATP, i.e., it can lock heat energy into the chemical form of energy for cellular functions.

## Introduction

In the past, there was a common belief that the living organisms on Earth could only utilize light energy and/or chemical energy, but not the environmental heat energy. Consequently, the life on Earth has been classified as two types based on their sources of energy: phototrophs and chemotrophs. Recently through bioenergetics elucidation studies with a new transmembrane electrostatic proton localization theory^1–7^ now called also as the transmembrane electrostatically localized protons (TELP) theory, Lee discovered that certain biosystems such as alkalophilic bacteria *Bacillus pseuodofirmus* are capable of utilizing environmental heat energy isothermally with TELP to help drive ATP synthesis^8–13^. This discovery indicated that the protonic bioenergetic systems may have a thermotrophic function that is able to isothermally generate significant amounts of Gibbs free energy from environmental heat (dissipated-heat energy)^8–14^. It naturally raises a fundamentally important question: Do the mitochondria-powered chemotrophs have a thermotrophic function featured with isothermal environmental heat energy utilization as well? The answer to this scientific question is now positive^10–13,15^.

This topic is related to Peter Mitchell’s chemiosmotic theory^16–18^, which we now know is not entirely correct so that it must be revised^2,3,5,7,8,13,19–22^. However, Mitchell’s equation for the “protonic motive force (pmf)” has entered many textbooks^23–26^ and is typically expressed as

1 $${\mathrm{pmf}}=\Delta\psi-\frac{2.3RT}{F} \Delta pH$$

Here, the pmf equation’s parameters are the transmembrane potential difference $\Delta\psi$, the gas constant *R*, the absolute temperature *T*, Faraday’s constant *F*, and the transmembrane bulk liquid-phase pH difference (∆*pH*) as defined previously^25,26^.

Recent studies^2,3,5,7,8,13,19–22^ showed that this Mitchellian textbook pmf equation (Eq. 1) could not really explain the bioenergetics in many biological systems. The most obvious evidences that clearly invalidate Mitchell’s pmf equation (Eq. 1) are the well-documented experimental observations of the alkalophilic bacteria (*Bacillus pseuodofirmus*)^27–29^ that keep “their internal pH about 2.3 units more acidic than the ambient bulk pH while ∆ψ is about 180 mV”^2,5,13,30–32^. In this well-documented case, the application of the Mitchellian textbook pmf equation (Eq. 1) would predict a pmf of only 44 mV that for decades could never really explain how the organisms can synthesize ATP with such a “small pmf”^2,5,33–35^.

As previously reported^5,7,13,20–22^, another deficiency of Mitchell’s chemiosmotic theory is its failure in addressing the legitimate question on whether the protonic coupling pathway for oxidative phosphorylation is localized at the membrane surface or delocalized throughout the bulk aqueous phase since 1961 when this question was first raised by Williams^36–43^. We now know, Mitchell's delocalized proton view cannot explain a number of well-documented experimental results including (but not limited to): (1) The “localized proton coupling characteristics” demonstrated in the “low salt” treated thylakoids of the well-documented Chiang-Dilley experiment^44^; (2) The observed mitochondrial “ΔpH surface component of pmf”^45^; (3) The “newly reported lateral pH gradient” along the mitochondrial inner membrane surface^46^; (4) An independent biomimetic study^47^ recently also showing a phenomenon of localized protons; and (5) The deficiency of Mitchell’s chemiosmotic theory that could not fully explain the energetics even in mitochondria and *E. coli* as recently noticed by Lee and other scientists^5,13,20,48^.

Furthermore, the novel transmembrane electrostatic proton localization (also called as TELP) theory^1,2,4–7,9,13^ developed recently with biomimetic experimental demonstrations^3,19,49,50^ showed how a “protonic capacitor” can form from excess protons at one side of a membrane with excess hydroxyl anions at the other side of the membrane. Through an effort in revising Eq. (1) to account for the TELP effects, the author has recently formulated a new pmf equation that showed to result in plenty of pmf for ATP synthesis in alkalophilic bacteria^2,51^. The newly developed pmf equation of Ref.^2^ was then applied to systematically elucidate the energetics in alkalophilic bacteria using the existing experimental data and a refined analysis for the cation exchange with TELP^10,12,52^. Surprisingly, large pmf values were found which substantially exceed the chemical energy upper limit of the redox-driven protonic pumps^10,11,14,15^. It was subsequently uncovered that the alkalophilic bacteria can effectively utilize the protonic thermal kinetic energy through “TELP at the liquid-membrane interface” in driving the synthesis of ATP molecules^8–11,13–15,53^.

Biomembrane including the mitochondrial membrane typically carries “negatively-charged surface groups” at its two sides attracting ions including cations and/or protons and thus forming “electrical double layers”^54^ as expected by the Gouy-Chapman theory^55^. As pointed out previously^5,7,13,22^, the membrane surface charges are “fixed” and their attracted ions including the associated electrical double layers are irrelevant to the membrane potential $\Delta\psi$ ; Consequently, the membrane-fixed surface-charges-attracted ions (including the electrical double layers) “do not contribute to the pmf”^56^ in living organisms. The membrane-fixed surface charge-associated “electrical double layers” are there all the time even before the biomembrane is physiologically energized and even when the cells including mitochondria are completely dead.

Our recent theoretical studies^5,7,22^ and experimental work^3,19,49,57^ showed a protonic capacitor: the formation of TELP demonstrated in an anode water-membrane-water cathode biomimetic system^19^. The experimental demonstration of the protonic capacitor provided a proof-of-principle for the creation of TELP, mimicking an energized biomembrane system such as a mitochondrion in its energized resting state^3^. With the TELP theory, we have successfully elucidated the bioenergetic significance in mitochondrial cristae formation^7^. The application of the TELP theory has now also resulted in a novel protonic action potential equation^22^ based on the protonic capacitor concept with deep insights for neuronal electrophysiology, that may constitute a complementary development to the classic Goldman–Hodgkin–Katz equation^22^.

Remarkably through our recent study^7^, we have now, for the first time, calculated the numbers of TELP (transmembrane electrostatically localized protons) in a range from 1.84 × 10^4^ to 7.36 × 10^4^ protons per mitochondrion, corresponding to a range of membrane potential $\Delta\psi$ from 50 to 200 mV for a mitochondrion with cristae^7^. This is a significant development since it may have important implications in helping address another problem of the Mitchellian chemiosmotic theory recently exposed by an independent study^58^, which finds “that there may be no more than seven H^+^ ions in total in the whole intermembrane space!” As pointed out by Bal et al.^58^, such a few (< 7) protons in the intermembrane/cristae space bulk liquid phase are obviously not sufficient to support the activities of ATP synthesis in a mitochondrion where “thousands of ATP synthase complexes are considered to be simultaneously active”^59^. We now understand, it is the large numbers of TELP (in a range from 1.84 × 10^4^ to 7.36 × 10^4^ protons) per mitochondrion that we have just recently calculated apparently play an essential role in helping driving ATP synthesis^7^.

More importantly as mentioned above, through the energetics analysis using the TELP theory^1,2,4–7,9,13^, we recently discovered that “the alkalophilic bacteria are not only chemotrophs, but also have a thermotrophic feature that can create significant amounts of Gibbs free energy through isothermal utilization of environmental heat energy with TELP (protonic thermal motion kinetic energy) to do useful work such as driving ATP synthesis”^13^.

What we recently discovered in the protonic energetics of alkalophilic bacteria^13^ is just a tip of the iceberg. We now understand that the thermotrophic feature may occur in many protonic bioenergetic systems including mitochondria, which produce the cellular fuel ATP to support complex life^60^. In the kingdom of animals including human, over 90% of cellular energy (ATP) is made by mitochondrial oxidative phosphorylation.

Therefore, in this paper, our updated pmf equation reported in the recent publications^2,5,7,13,51^ is now further extended systematically to calculate the protonic motive force (pmf) and to better elucidate the energetics in mitochondria, employing our newly determined cation-proton exchange equilibrium constants^3^ for sodium, potassium and magnesium, and utilizing the published animal mitochondria experimental data^61^.

The study reported in this article is timely, especially, since it has recently been recognized that Mitchell’s chemiosmotic theory could hardly explain the bioenergetics in mitochondria^11,15,48^. For example, Silverstein (2014) noticed that the pmf calculated from the Mitchellian pmf equation (Eq. 1) would not be enough to drive ATP synthesis when the mitochondrial membrane potential is around 80 mV^48^. Another independent study by Nath^62^ also encountered the problems of the Mitchellian chemiosmotic theory being “inadequate” to explain ATP synthesis in biological systems^63–66^ and thus proposed an alternative theory of energy transduction^67^. The mitochondrial membrane potentials in living cells are now known to be mostly about 56 mV, 105 ± 0.9 mV and 81 ± 0.7 mV^68^, 91 ± 11 mV and 81 ± 13 mV^69^, and also 114 mV^70^ and 123 mV^71^. That is, Mitchell’s chemiosmotic theory and its associated textbook pmf equation (Eq. 1) is not able to explain the protonic energetics even in mitochondria! This energetic problem of paramount importance will be addressed here in this article employing the TELP theory^1,2,4–7,9,13^ with new discovery in analyzing the protonic motive force (pmf) including the local pmf. Note, pmf can be translated to Gibbs free energy change $\Delta G$ according to a simple relation ($\Delta G=-F{\mathrm{pmf}})$ with the Faraday constant ($F$). Therefore, the study reported here may represent not only new progress in protonic bioenergetics, but also the latest discovery on a naturally occurring novel energy phenomenon: the thermotrophic function featured as isothermal environmental heat energy utilization with TELP to do useful work in driving ATP synthesis that we now know to occur in the mitochondria of our body as well.

## Methods

### Newly formulated protonic motive force equations with TELP

The bioenergetic process for oxidative phosphorylation (ATP synthesis) in mitochondria comprises the steps of creating an excess number of protons on the intermembrane space/cristae side of the mitochondrial inner membrane accompanied by a corresponding number of hydroxyl ions on the matrix side^5,13^ typically, through the respiratory redox-driven electron-transport-coupled proton pumps across the mitochondrial inner membrane^7^. According to the TELP theory^1,2,4–7,9,13,22^, the excess positively charged protons in the aqueous liquid on the intermembrane space/cristae side of the mitochondrial inner membrane will electrostatically become localized at the liquid-membrane interface, attracting an equal number of excess negatively charged hydroxyl anions to the other side (matrix) of the mitochondrial inner membrane to form a “protons-membrane-anions capacitor structure”. This theory is built on the fundamental understanding that liquid water can act as a protonic conductor, which is well in line with the knowledge that protons quickly transfer among water molecules by the “hops and turns” mechanism first outlined by Grotthuss^72–75^.

Therefore, a newly formulated equation for the protonic motive force (pmf) across a biomembrane considering TELP was introduced more recently through the author’s latest publications^5,7,13,22^ as

2 $${\mathrm{pmf}}=\Delta\psi+\frac{2.3RT}{F}{\mathrm{log}}_{10}\left(\left[{H}_{pB}^{+}\right]/\left[{H}_{nB}^{+}\right]\right)+\frac{2.3RT}{F}{\mathrm{log}}_{10}\left(1+\left[{H}_{L}^{+}\right]/\left[{H}_{pB}^{+}\right]\right)$$

Here $\Delta\psi$ is the “membrane potential from the *p*-side to the *n*-side” as defined by Mitchell^76,77^, Nicholls and Ferguson^25,26^; $\left[{H}_{L}^{+}\right]$ is the TELP concentration at the liquid-membrane interface on the positive (*p*) side of the membrane; $\left[{H}_{pB}^{+}\right]$ is the “proton concentration in the bulk aqueous *p*-phase” (intermembrane space in the case of mitochondria); and $\left[{H}_{nB}^{+}\right]$ is the “proton concentration in the bulk liquid *n*-phase” (matrix in mitochondria)^5^. The first two terms of Eq. (2) comprise the “Mitchellian bulk phase-to-bulk phase proton electrochemical potential gradients” that we now call as the “classic” pmf, equivalent to that of Eq. (1); whereas the last term accounts for the “local” pmf from TELP at the liquid-membrane interface^7,13^.

For a protonic capacitor, as reported previously^2,5,7,13,22,51^, the ideal TELP concentration ${[{H}_{L}^{+}]}^{0}$ on the positive (*p*) side of the membrane is a function of the transmembrane potential $\Delta\psi$ as expressed in the following equation:

3 $${[{H}_{L}^{+}]}^{0}=\frac{C}{S}\cdot\frac{\Delta\psi}{l\cdot F}$$

where $C/S$ is “the specific membrane capacitance per unit surface area”, $l$ is “the thickness of the localized proton layer”^22^.

In mitochondria, non-proton cations in the bulk aqueous liquid phase can exchange with TELP at the liquid-membrane interface and thereby reduce TELP concentration. To account for the exchanging effect, this study employed the following equation developed recently also by Lee for the steady-state TELP concentration $\left[{H}_{L}^{+}\right]$ in consideration of cation-proton exchange with each of the cation species ${M}_{pB}^{i+}$ of the bulk liquid *p*-phase at the equilibrium state as reported in the recent publications^2,5,7,13,22,51^,

4 $$\left[{H}_{L}^{+}\right]=\frac{{\left[{H}_{L}^{+}\right]}^{0}}{\prod_{i=1}^{n}{\{K}_{Pi}\left(\frac{\left[{M}_{pB}^{i+}\right]}{\left[{H}_{pB}^{+}\right]}\right)+1\}}$$

Here $\left[{M}_{pB}^{i+}\right]$ is the non-proton cation concentration in the bulk liquid *p*-phase, and ${K}_{Pi}$ is the equilibrium constant for the cation to exchange with TELP. The equilibrium constant ${K}_{Pi}$ is defined (and can be experimentally measured) as the ratio of the delocalized proton concentration $\left[{H}_{pB}^{+}\right]$ to the cation concentration $\left[{M}_{pB}^{i+}\right]$ in the bulk aqueous *p*-phase when the cation-proton exchanging process reaches the midpoint at an equilibrium state where the steady-state TELP concentration $\left[{H}_{L}^{+}\right]$ is equal to the localized cation concentration $\left[{M}_{pL}^{i+}\right]$ at the liquid-membrane interface^3^.

Note, each of the physical quantities appearing in Eqs. (2−4) may, in principle, be determined through experimental measurements. There are “no freely adjustable parameters”. The calculations with Eq. (3) here used “$C/S$ = 13.2 mf/m^2^ as an averaged membrane capacitance based on measured experimental data”^78^ and “$l$ = 1 nm as a reasonable thickness of TELP layer” as previously explained in Ref.^2,5,7,13,19,22^.

In the work reported in the next section, the TELP bioenergetics analysis with Eqs. (2−4) is extended to mitochondria not only by making a better treatment for cation-proton exchange^3,49^, but also by incorporating this treatment to calculate the total pmf (including both the “classic” and “local” pmf values) for the measured values for $\Delta\psi$, $\left[{H}_{pB}^{+}\right]$, and $\left[{H}_{nB}^{+}\right]$ using the well-documented animal mitochondria experimental data^61^.

### Cation exchange reduction factors on TELP

In the experimental study of “mitochondrial ATP-ADP exchange” by Chinopoulos et al.^61^, “mitochondrial membrane potential $\Delta\psi$ was measured in a range from 60 to 160 mV using fluorescence quenching with a cationic dye owing to its accumulation in energized mitochondria”. The Chinopoulos et al. experimental results demonstrated the synthesis of ATP that was measured as the “ATP efflux rate” at a membrane potential $\Delta\psi$ as low as 60 and 80 mV. Furthermore, their experimental work^61^ also demonstrated that “there is essentially no or little bulk-phase pH difference” between the matrix and the intermembrane space: the “∆pH_max_ is only ~ 0.11”. That is, under the given reaction medium pH 7.25 (${pH}_{pB}$), mitochondria matrix pH was about 7.35 (${pH}_{nB}$) during the state three. Another independent study^79^ also consistently showed that the mitochondria matrix pH is about 7.3, which essentially is identical to that of the cytosol. These experimental observations are well corroborated with the Lee team’s “experimental results from a biomimetic anode water-membrane-water cathode system where the bulk-phase liquid pH in the anode liquid chamber was observed to be essentially about the same as that in the cathode liquid chamber before and after energization by excess protons at one side of the membrane and excess hydroxyl anions at the other side”^3,8,19,49^. Therefore, the “measured experimental parameters (data) of the reaction medium pH 7.25 (${pH}_{pB}$) and mitochondria matrix pH 7.35 (${pH}_{nB}$) during the state three as reported by Chinopoulos et al.”^61^ are employed in the biophysical analyses here as previously reported^5,7^.

The concentrations of the bulk liquid-phase cations Na^+^, K^+^, and Mg^2+^ in the mitochondria are listed in Table 1 (adapted from Ref.^5^). As recently reported^5,7^, Table 1 presents the value for each of the cation-proton exchange constants ${K}_{Pi}$ for Na^+^, K^+^, and Mg^2+^ used in the calculation. More importantly, it shows the “calculated cation exchange reduction factors of TELP concentration for ${pH}_{pB}$ = 7.25, which was the liquid medium pH used in the mitochondrial membrane potential ($\Delta\psi$) measurement experiment”^61^. It also shows that “the total product of these factors (total cation exchange reduction factor in the denominator of Eq. (4)) is 1.29, which is close to one, indicating a relatively small role of cation-proton exchange in modulating TELP concentration at the liquid-membrane interface in mitochondria”^5,7^.

**Table 1:** The total product (1.29) of cation exchange reduction factors calculated from experimental cation concentrations in the bulk liquid reaction medium for mitochondria (as reported in Ref.^5,7,61^), their associated cation-proton exchange equilibrium constants, and cation exchange reduction factors for TELP concentration at the liquid-membrane interface with the bulk liquid reaction medium ${pH}_{pB}$ = 7.25. Adapted from Ref.^5^.

Cation species ${M}_{pB}^{i+}$	Cation species concentration $\left[{M}_{pB}^{i+}\right]$	Exchange equilibrium constant ${K}_{Pi}$	${K}_{Pi}\left(\frac{\left[{M}_{pB}^{i+}\right]}{\left[{H}_{pB}^{+}\right]}\right)+1$
Na^+^	10 mM	5.07 × 10^–8^	1.01
K^+^	128 mM	6.93 × 10^–8^	1.16
Mg^++^	1.0 mM	5.42 × 10^–6^	1.10
		**Total product of cation exchange reduction factors:** $\prod_{i=1}^{n}{\{K}_{Pi}\left(\frac{\left[{M}_{pB}^{i+}\right]}{\left[{H}_{pB}^{+}\right]}\right)+1\}$	**1.29**

## Results and discussions

### Contribution from TELP to mitochondrial pmf

**Figure 1:**
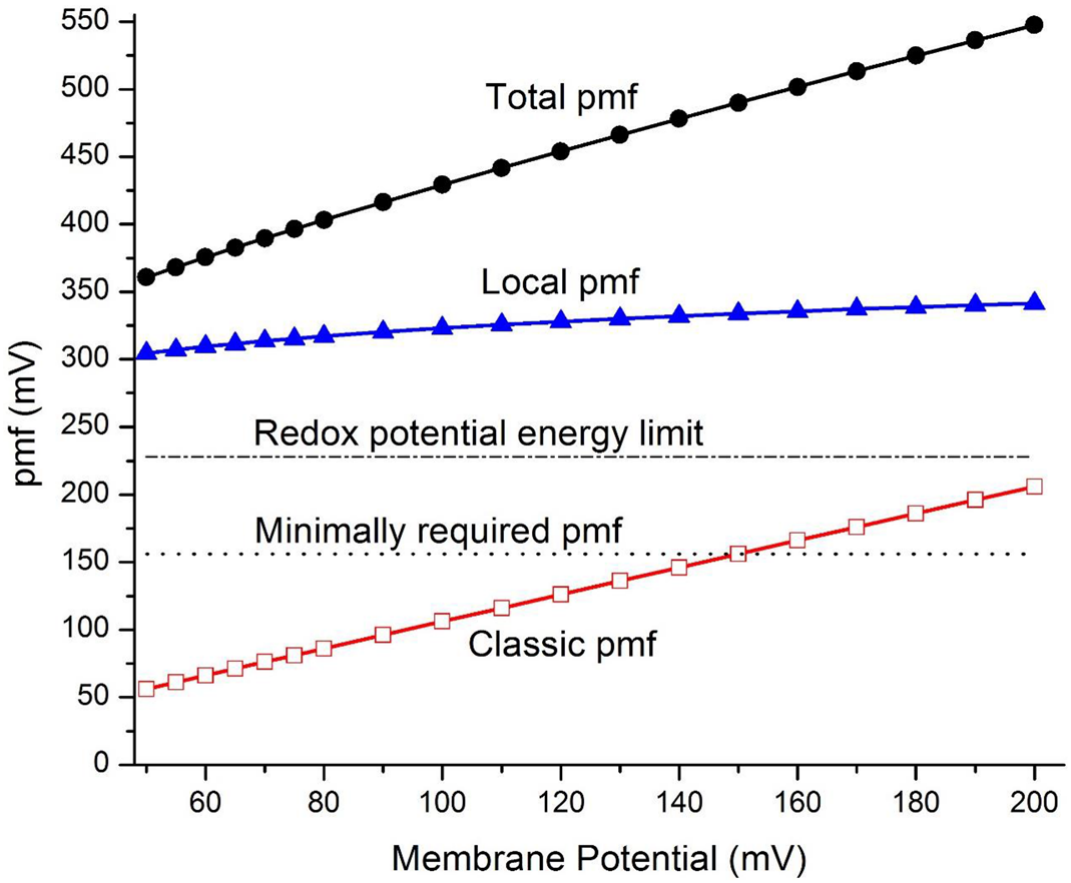
The values of total pmf, local pmf and classic pmf in mitochondria calculated as a function of membrane potential $\Delta\psi$ using the newly formulated pmf expression (Eqs. 2−4) in comparison with the minimally required pmf (156 mV) for ATP synthesis and with the redox potential chemical energy upper limit (228 mV).

Transmembrane electrostatically localized protons (TELP) significantly contribute to the total protonic motive force (pmf) that can be translated to Gibbs free energy change ($\Delta G=-F{\mathrm{pmf}})$. Table 2 lists the values of mitochondrial pmf and the associated properties calculated as a function of transmembrane electrical potential difference $\Delta\psi$ using Eqs. (2−4) under the given reaction medium pH 7.25 (${pH}_{pB}$), mitochondria matrix pH 7.35 (${pH}_{nB}$) and taking cation-proton exchange into account as described above. The calculated pmf as a function of the transmembrane potential $\Delta\psi$ is displayed in Fig. 1, in comparison with the classic and local pmf contributions. These results showed that TELP dominantly contribute to the overall strength of pmf.

**Table 2:** Mitochondrial protonic motive force (pmf) and the associated properties including local pmf, calculated as a function of membrane potential $\Delta\psi$ using Eqs. (2−4) based on the measured properties (${pH}_{pB}$, ${pH}_{nB},$ $\Delta\psi )$ with the known reaction medium compositions of ref.^61^. The cation concentrations, proton-cation exchange equilibrium constants and cation exchange reduction factor (1.29) are from Table 1; and the temperature *T* = 310 K. The “local” pmf is the last term in Eq. (2), while the first two terms of Eq. (2) give the “classic” Mitchellian pmf. Adapted and updated from Ref.^5^.

$\Delta\psi$ (mV)	${pH}_{pB}$	${pH}_{nB}$	${[H}_{L}^{+}]$ ^0^(mM)	Exchange reduction factor	${[H}_{L}^{+}]$ (mM)	Local pmf (mV)	Classic Pmf (mV)	Total pmf (mV)	Local pmf/classic pmf
50	7.25	7.35	6.84	1.29	5.30	306	56	362	5.44
55	7.25	7.35	7.52	1.29	5.83	308	61	369	5.04
60	7.25	7.35	8.21	1.29	6.36	311	66	377	4.69
65	7.25	7.35	8.89	1.29	6.89	313	71	384	4.39
70	7.25	7.35	9.58	1.29	7.42	315	76	391	4.13
75	7.25	7.35	10.3	1.29	7.95	316	81	398	3.90
80	7.25	7.35	10.9	1.29	8.48	318	86	404	3.69
90	7.25	7.35	12.3	1.29	9.55	321	96	417	3.34
100	7.25	7.35	13.7	1.29	10.6	324	106	430	3.05
110	7.25	7.35	15.0	1.29	11.7	327	116	443	2.81
120	7.25	7.35	16.4	1.29	12.7	329	126	455	2.61
130	7.25	7.35	17.8	1.29	13.8	331	136	467	2.43
140	7.25	7.35	19.2	1.29	14.8	333	146	479	2.28
150	7.25	7.35	20.5	1.29	15.9	335	156	491	2.15
160	7.25	7.35	21.9	1.29	17.0	337	166	503	2.03
170	7.25	7.35	23.3	1.29	18.0	338	176	514	1.92
180	7.25	7.35	24.6	1.29	19.1	340	186	526	1.83
190	7.25	7.35	26.0	1.29	20.2	341	196	537	1.74
200	7.25	7.35	27.4	1.29	21.2	343	206	549	1.66

A pmf of at least 156 mV is required to overcome the mitochondrial phosphorylation potential of − 416 mV (− 9.6 × 4.187 ÷ $F$) as calculated from the Gibbs free energy change (ΔG_ATP_) of + 9.6 kcal/mol reported in Ref.^48,80,81^ for ATP synthesis through oxidative phosphorylation with a proton-to-ATP ratio of 8/3 (416 mV/2.67 = 156 mV). The proton-to-ATP ratio of 8/3 is consistent with the animal mitochondrial F_0_F_1_-ATP synthase structure, which has 3 catalytic sites for ATP synthesis, driven by a flow of 8 protons per revolution through the 8 c-subunits in its rotary molecular machinery^60,82–84^.

In consideration of an additional proton being used in the transport of Pi and ADP into, and of ATP out of, the mitochondrial matrix, the overall H^+^/ATP stoichiometry is increased by 1 to account for the additional proton (2.67 + 1 = 3.67). If the mitochondrial phosphorylation potential is conservatively assumed as − 416 mV (translating to − 40.2 kJ mol^−1^) for both ATP synthesis and transport together, then the minimally required pmf would be 113 mV (416 mV/3.67). According to a previous study in functional animal heart cells^85^, the critical ATP hydrolysis free energy is approximately − 63.5 kJ mol^−1^, which may be translated to a phosphorylation potential of − 658 mV (− 63.5 kJ mol^−1^/$F$). Accordingly, the minimally required pmf for ATP synthesis and transport together should be 179 mV (658 mV/3.67) in a functional animal cell.

Note, the phosphorylation potential for ATP synthesis used by Slater^81^ for his 1967 evaluation of the Mitchellian chemiosmotic hypothesis is + 15.6 kcal/mol that was measured by Cockrell et al.^80^ in isolated rat liver mitochondria. Remarkably, this phosphorylation potential of + 15.6 kcal/mol (translating to 65.3 kJ mol^−1^) for ATP synthesis is quite close to the magnitude of the critical free energy − 63.5 kJ mol^−1^ for ATP hydrolysis in a functional animal heart cell obtained by Wu et al.^85^. According to this phosphorylation potential of + 15.6 kcal/mol (translating to − 65.3 kJ mol^−1^ ÷$F$  =  − 677 mV), the minimally required pmf for ATP synthesis in mitochondria should be 254 mV (677 mV/2.67) while the minimally required pmf for ATP synthesis and transport out of mitochondria would be 184 mV (677 mV/3.67).

To give the most benefits of doubts for the Mitchellian chemiosmotic theory and its associated textbook pmf equation (Eq. 1), we will use the minimally required pmf of 156 mV for ATP synthesis based on the conservative value of + 9.6 kcal/mol (translating to 40.2 kJ mol^−1^)^48^ for the phosphorylation potential of ATP synthesis in the following analysis. As shown in Fig. 1, the total pmf including the contribution from TELP is well above the minimally required value of 156 mV to synthesize ATP, while the Mitchellian pmf is below this minimally required pmf at any of the $\Delta\psi$ values below 150 mV. This result indicates that the classic Mitchellian chemiosmotic theory could hardly explain the energetics even in mitochondria as noticed previously also by an independent study^48^. The mitochondria membrane potentials in a range from 40 to 140 mV have been determined in vivo in living human fibroblast cells with the techniques of fluorescence microscopic imaging and deconvolution^68^. It is now quite clear that the classic pmf alone cannot explain how the living cells are able to synthesize ATP and grow; In contrast, the synthesis of ATP and cell growth can now be well explained by the total pmf (Fig. 1) as calculated according to the TELP theory^1,2,4,5,7,9,13^.

Based on the in vivo measurement by Zhang et al.^68^ using “fluorescent dye tetramethylrhodamine methyl ester” (which equilibrates between mitochondria and cytosol in living cells), the distribution of the mitochondrial membrane potentials in fibroblasts was determined to be mostly about 56 mV when the mean cytosolic dye fluorescent intensity was used as a threshold. With a somewhat higher threshold (“mean + SD”) in their analysis^68^, the mitochondrial membrane potential ($\Delta\psi$) values in fibroblasts and “N2a” cells were reported to be 105 ± 0.9 mV and 81 ± 0.7 mV, respectively. At any of these reported mitochondrial membrane potential ($\Delta\psi$) values (56 mV, 105 ± 0.9 mV, and 81 ± 0.7 mV), the classic pmf value as presented in Fig. 1 is below the minimally required pmf value of 156 mV to drive ATP synthesis. That is, the “∆pH_max_ is only ~ 0.11” observed by Chinopoulos et al.^61^ can be translated to a delocalized pmf of no more than 6.8 mV; with these low mitochondrial membrane potential ($\Delta\psi$) values, the total classic pmf values are no more than 63 mV, 112 ± 0.9 mV, and 88 ± 0.7 mV, which are all below the minimally required pmf of 156 mV to drive ATP synthesis. Therefore, the Mitchellian chemiosmotic theory and its pmf equation (Eq. 1) clearly failed to explain how the mitochondria can make ATP through oxidative phosphorylation to support the cell growth here.

An independent experimental study by Akhmedov et al.^86^ showed that “the resting matrix pH was only 0.19 pH units higher than the cytosolic pH”, which is remarkably similar to the observation of “∆pH_max_ is only ~ 0.11” by Chinopoulos et al.^61^. If this ∆pH of 0.19 (translating to a delocalized pmf of 12 mV) is used to calculate pmf (with the mitochondrial membrane potential ($\Delta\psi$) values of 56 mV, 105 ± 0.9 mV, and 81 ± 0.7 mV), the total classic pmf values would then be 68 mV, 117 ± 0.9 mV, and 93 ± 0.7 mV, all of which are also below the minimally required pmf value of 156 mV to drive ATP synthesis. Again, the Mitchellian chemiosmotic theory and its pmf equation (Eq. 1) clearly failed to explain how the mitochondria can make ATP through oxidative phosphorylation to support the cell growth here.

Akhmedov et al.^86^ further showed that “during glucose stimulation (increasing the glucose concentration from 2.5 to 16.7 mM), mitochondrial matrix pH in the insulin-secreting INS-1E cells increased from pH 7.25 ± 0.04 to 7.78 ± 0.02” and “fifteen minutes after initiation of the glucose response, the ∆pH increased 3.5-fold to 0.66”. If this high glucose-stimulated $\Delta \mathrm{p}\mathrm{H}$ of 0.66 (translating to a delocalized pmf of 40 mV) is used with the mitochondrial membrane potential ($\Delta\psi$) values (56 mV, 105 ± 0.9 mV, and 81 ± 0.7 mV), the total classic pmf values would then be 106 mV, 145 ± 0.9 mV, and 121 ± 0.7 mV; all of them are still below the minimally required pmf value of 156 mV to drive ATP synthesis. Therefore, once again, the classic Mitchellian chemiosmotic theory and its pmf equation (Eq. 1) again failed to explain how the mitochondria can make ATP to support the cell growth.

These findings are well corroborated with the mysterious problem previously noticed by Silverstein (2014) as a “thermodynamic efficiency of 113%” in mitochondria at a membrane potential of around 80 mV^48^. According to the classic pmf equation (Eq. 1), to avoid the “impossibly high efficiency (> 100%)” for mitochondria, one would have to “adjust” the bulk-phase “ΔpH (in–out)” to an arbitrary value of at least “ + 2.5”. However, it is now quite clear that the bulk-phase ΔpH (in–out) is nearly zero: “ΔpH_max_ is only ~ 0.11” based on the modern experimental measurements^61^ and modeling analysis of mitochondria^87^. The observed bulk-phase ΔpH of nearly zero in mitochondria^61^ is also corroborated with the prediction from the transmembrane electrostatic proton localization theory^2,5,7–9^ with the understating that mitochondrial inner membrane is rather impermeable to ions^88,89^. Another independent study using a pH-sensitive GFP^90^ has recently also showed “that the intracristae lumen does not provide a reservoir for substrate protons for ATP synthesis” indicating “kinetic coupling of the respiratory chain with ATP synthase, but not proton gradients, drives ATP production in cristae membranes”.

Note, the data in Table 2 and Fig. 1 show that the classic pmf value would be above the minimally required pmf of 156 mV only if the mitochondrial membrane potential is above 150 mV. In some of the recent literatures^91^, the bulk-liquid phase $\Delta \mathrm{p}\mathrm{H}$ across the mitochondrial inner membrane was believed to be typically less than 0.5 unit, such as “the resting matrix pH (~ 7.6) and ΔpH_m_ (~ 0.45) of HeLa cells at 37 °C” reported by Poburko et al.^92^. In that case, only when the mitochondrial membrane potential is above 130 mV, the classic pmf value would be above the minimally required pmf (156 mV). This is an interesting feature, since it can probably explain why it took so long in the last 50 years for the scientific community to fully realize the problems with the Mitchellian chemiosmotic theory including its pmf equation (Eq. 1). For example, like in the parable of “the blind men and an elephant” where a blind man touching the side of an elephant body may passionately feel the elephant “is a wall”, some of the readers especially those who may have been fully occupied by the classic Mitchellian chemiosmotic theory looking at these classic pmf data points above the 156 mV level (Table 2 and Fig. 1) such as a classic pmf value from 186 to 196 mV at a membrane potential ($\Delta\psi$) from 180 to 190 mV previously reported in isolated mitochondria^93,94^ could still feel hard to judge whether the Mitchellian chemiosmotic theory really has deficiencies or not.

We now know that, even at the high membrane potentials ($\Delta\psi$) such as 190 and 200 mV, its associated classic pmf value 196 and 206 mV as shown in Table 2 actually are still below the minimally required pmf of 254 mV (677 mV/2.67) for ATP synthesis in mitochondria, according to the phosphorylation potential of + 15.6 kcal/mol (translating to − 677 mV) used by Slater^81^.

Anyhow, a good theory should be able to explain the bioenergetics with a full range of scientific data, for example, in the entire range of mitochondrial membrane potential ($\Delta\psi$) values from 50 up to 200 mV, not just at the high membrane potential above 150 mV. Furthermore, the in vivo mitochondrial membrane potential ($\Delta\psi$)^70,95,96^ values are mostly below 150 mV including 56 mV, 105 ± 0.9 mV, and 81 ± 0.7 mV reported by Zhang et al.^68^, 91 ± 11 mV and 81 ± 13 mV measured by Gurm et al. (2012) using the techniques of 4-[^18^F]fluorophenyltriphenylphosphonium and in vivo positron emission tomography (PET) measurement^69^, and also 114 mV and 123 mV measured in swine and human respectfully using an improved PET-based method by Alpert et al.^70^ and by Pelletier-Galarneau et al.^71^. We now understand that the classic Mitchellian chemiosmotic theory and its pmf equation (Eq. 1) cannot explain the mitochondrial energetics of oxidative phosphorylation in living cells because it fatally misses to account for the pmf contribution from TELP^5,7,13^.

As shown in Table 2 and Fig. 1, the local pmf from TELP was calculated to be a range from 306 to 343 mV, which is a function of the membrane potential ($\Delta\psi$) in a range from 50 up to 200 mV. The total pmf is the sum of the classic pmf and local pmf, which in this case is in a range from 362 to 549 mV. The use of the local pmf (and the total pmf) can now easily explain how mitochondria are able to synthesize ATP since they are all well above the minimally required pmf of 156 mV at any of the mitochondrial membrane potential ($\Delta\psi$) values in the entire range from 50 to 200 mV. Thus, the newly formulated pmf expression in Eqs. (2−4) consistently provides an excellent elucidation for the energetics of oxidative phosphorylation in mitochondria without requiring any arbitrary adjustment in the number of the bulk-phase “ΔpH (in–out)” that the previous study^48^ with the classic Mitchellian doctrine had to require.

**Figure 2:**
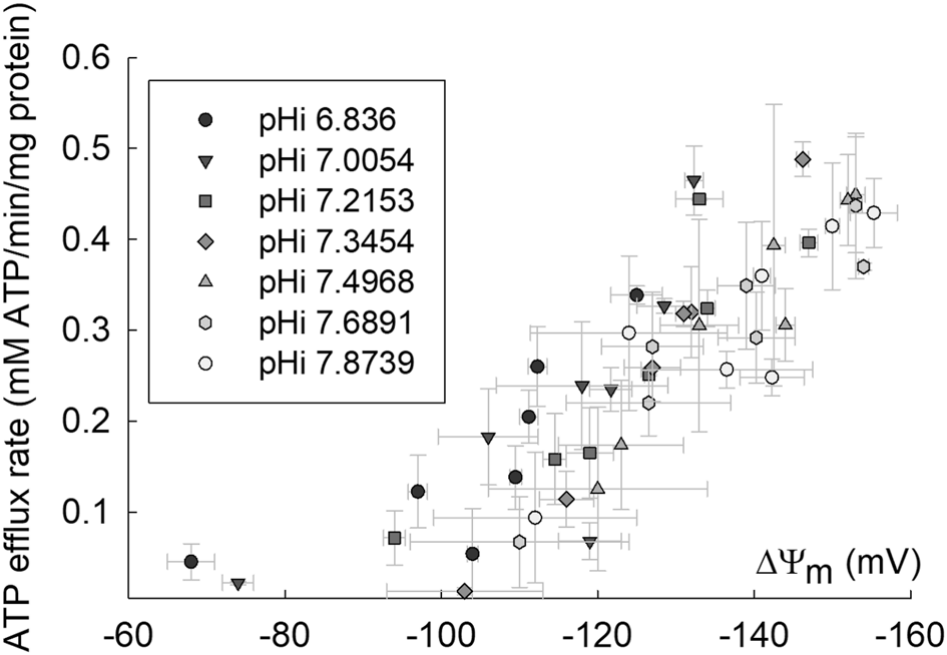
ATP production rate in isolated mitochondria experimentally measured as the steady-state ATP efflux rate mediated through the adenine nucleotide translocase (ANT) with mitochondrial membrane potential ($\Delta\psi$) manipulated to various values in a range from 60 to 160 mV at various matrix pH (pHi) values in isolated rat mitochondria energized with “5 mM K-glutamate and 5 mM K-malate” in the presence of dissolved O_2_. Adapted from Chinopoulos et al.^61^.

This new elucidation for the energetics in mitochondria can now also be well corroborated with the experimental results from the measurements of ATP production as a function of membrane potential ($\Delta\psi$) during the state 3 after the addition of ADP in isolated rat mitochondria under oxidative phosphorylation experimental conditions^61^. Figure 2 presents the ATP production rate measured as the steady-state ATP efflux rate mediated through the adenine nucleotide translocase as a function of mitochondrial membrane potential ($\Delta\psi$) manipulated through titration using uncoupler SF 6847 during the state 3 to various $\Delta\psi$ values in a range from 60 to 160 mV at various matrix pH (pHi) values in isolated rat mitochondria energized with 5 mM K-glutamate and 5 mM K-malate in the presence of dissolved O_2_, as reported previously in great details^61^. As shown in Fig. 2, the oxidative phosphorylation ATP production through the activity of mitochondrial ATP synthase is indeed still going on even in the low membrane potential range (60–150 mV) as predicted by the newly calculated pmf data (Table 2 and Fig. 1) using the newly formulated pmf equations (Eqs. 2−4) where the total pmf values owning to the effect of TELP are still well above the minimally required pmf of 156 mV for ATP synthesis. Furthermore, the observed pattern of the ATP efflux rate which decreases as mitochondrial membrane potential ($\Delta\psi$) is reduced (Fig. 2) is generally also in agreement with the pattern of the total pmf including the local pmf that is a function of the membrane potential ($\Delta\psi$) as shown in Fig. 1.

Under the experimental conditions of Fig. 2, based on the known metabolic pathways^97–101^, the conversion of a fed glutamate molecule to α-ketoglutarate and finally to oxaloacetate through part of the tricarboxylic acid (TCA) cycle in mitochondria could produce 1 FADH_2_ plus 3 NADH (1 NADH from glutamate to α-ketoglutarate and 2 NADH from α-ketoglutarate to oxaloacetate) and an ATP by substrate-level phosphorylation (at the step from succinyl-CoA to succinate), while the use of a fed malate molecule to oxaloacetate with the TCA cycle can produce at least 1 NADH. The use of 4 NADH and 1 FADH_2_ through the mitochondrial oxidative phosphorylation process could synthesize and transport 12.5 ATP [(4 NADH × 10 protons/NADH + 1 FADH × 6 protons/FADH_2_) / (3.67 protons/ATP)] out of mitochondria via the adenine nucleotide translocase. That is, the measured steady-state ATP efflux rate (Fig. 2) apparently is contributed mainly by the ATP production from the oxidative phosphorylation process. Merely a small fraction (1/13.5 × 100% = 7.4%) of the ATP production could be from the substrate-level phosphorylation of the TCA cycle, although the percentage from the substrate-level phosphorylation could be higher than 7.4% when the mitochondrial membrane potential ($\Delta\psi$) is reduced to an extremely low level (near or below 60 mV) by titration using large amounts of uncoupler SF 6847.

Therefore, the newly calculated pmf data including the local pmf (Table 2 and Fig. 1) in conjunction with the experimentally measured ATP production (Fig. 2) have now clearly showed that ATP synthesis through oxidative phosphorylation by mitochondrial ATP synthase can indeed occur at a low membrane potential ($\Delta\psi$) anywhere in a range from about 60 to 150 mV. This finding is remarkably in line with the independent observations of mitochondrial membrane potentials in living cells being mostly about 56 mV, 105 ± 0.9 mV and 81 ± 0.7 mV^68^, 91 ± 11 mV and 81 ± 13 mV^69^, and also 114 mV^70^ and 123 mV^71^ where apparently significant amounts of ATP are synthesized at such low mitochondrial membrane potentials to support the growth and activities of the living cells. That is, this new understanding may have major scientific implications with significant insights in explaining how ATP can be synthesized through mitochondrial ATP synthase with the effect of TELP at such remarkably low mitochondrial membrane potentials ($\Delta\psi$ about 56 mV, 105 ± 0.9 mV and 81 ± 0.7; 91 ± 11 mV and 81 ± 13 mV, and also 114 mV and 123 mV) in the living cells^68–71^.

### TELP utilizing mitochondrial environmental heat energy for ATP synthesis: a thermotrophic feature?

As presented in Table 2 and Fig. 1, all the total pmf values calculated through Eqs. (2−4) for mitochondria are well above the minimally required pmf value for ATP synthesis; Thus, the ATP synthesis in mitochondria can now be well explained for the entire range of membrane potential ($\Delta\psi$) from 50 to 200 mV. However, it is surprising that many of these pmf values (such as 362, 369, 391, 404, 430 and 455 mV) are significantly larger than the “redox potential energy pmf upper limit (228 mV)” that could be maximally accounted for by “the redox-driven protonic pump system” based on the redox potential difference (about 1140 mV) “between the electron donor NADH (E_m,7_ =  − 320 mV) to the terminal electron acceptor O_2_ (E_m,7_ =  + 820 mV)”^26^ as analyzed previously in regarding to the redox potential chemical energy limit of 228 mV^13^.

Note, this redox potential chemical energy upper limit for the pmf of 228 mV is still smaller than the minimally required pmf of 254 mV (677 mV/2.67) for ATP synthesis in mitochondria, according to the mitochondrial phosphorylation potential of + 15.6 kcal/mol (translating to − 677 mV)^81^. Therefore, it also indicates that there must be another disparate energy mechanism (which we now know is the thermotrophic feature associated with TELP herein) to explain the mitochondrial energetics of oxidative phosphorylation.

How could the total transmembrane pmf exceed the mitochondrial chemical energy upper limit (228 mV)? We now understand that mitochondria can isothermally utilize the environmental heat energy (also known as the temperature-dependent molecular thermal motion kinetic energy) associated with TELP in “driving the synthesis of ATP from ADP and Pi through F_0_F_1_-ATP synthase”. Therefore, mitochondria represent not only a chemotrophic system but also have a significant thermotrophic feature.

As recently reported^13^, the formation of a TELP layer apparently constitutes some kind of “negative entropy effect”^8,9,13^ that “brings the excess protons to the mouths of the pmf users (F_0_F_1_-ATP synthase) where the protons can isothermally utilize their molecular thermal motions (protonic thermal kinetic energy *k*_*B*_*T*) possibly including their random and chaotic Brownian motions to push through the doors of F_0_F_1_-ATP synthase in driving ATP synthesis”. That is, although the thermal energy-associated protonic translational motions (kinetic energy) are random and chaotic in all directions, “a localized proton at the water-membrane interface” has a much higher probability to chaotically hit through the mouth (F_0_ protonic channel) of F_0_F_1_-ATP synthase in driving the “F_0_ rotary molecular machinery for ATP synthesis” (thus the protonic thermal motion kinetic energy may be utilized) than “a delocalized proton in the bulk liquid phase that is far away from the protonic users”. As shown with the third term in Eq. (2) “for the local pmf”, the thermotrophic function featured as “the utilization of protonic thermal kinetic energy *k*_*B*_*T*” is essentially expressed as “RT (= *k*_*B*_*T* ⋅ *N*_A_) which equals to the product of the Boltzmann constant *k*_*B*_, the mitochondrial temperature *T* and the Avogadro constant *N*_A_”.

The “delocalized protons” in the mitochondrial cristae bulk liquid phase “are quite far away from the membrane surface”; Thus, the random and chaotic thermal motions of the delocalized protons in the bulk liquid phase are “not within the striking distance for them to hit into the F_0_F_1_-ATP synthase protonic channel to drive the rotary molecular machinery for ATP synthesis”. As discussed also in the recent publication^13^, “the delocalized protons could also do the work when they are at the liquid-membrane interface near the F_0_F_1_-ATP synthase”. Therefore, “the thermal energy factor RT (= *k*_*B*_*T* ⋅ *N*_A_)” is also in the second term of Eq. (2). However, the value of ${\mathrm{log}}_{10}\left(\left[{H}_{pB}^{+}\right]/\left[{H}_{nB}^{+}\right]\right)$ was nearly zero in the case of mitochondria; consequently, the delocalized protons in mitochondria did not significantly contribute to the thermotrophic feature in driving ATP synthesis here.

As pointed out in the recent publication^13^, “the protonic bioenergetics systems operate widely in nearly all organisms known today”. Therefore, this special thermotrophic function associated with TELP “has occurred probably for billions of years already on Earth”. As presented in Fig. 1, the amount of local pmf as calculated according to the third term of Eq. (2) quantitatively represents the activity of this amazing thermotrophic function isothermally utilizing environmental heat energy associated with TELP. That is, the following local pmf equation has fundamental scientific significance in relation to the thermotrophic function featured as the “isothermal environmental heat energy utilization” with TELP.

5 $${\mathrm{local}}{\mathrm{pmf}}=\frac{2.3RT}{F}{\mathrm{log}}_{10}\left(1+\left[{H}_{L}^{+}\right]/\left[{H}_{pB}^{+}\right]\right)$$

According to Eq. (5), this special protonic thermotrophic function is mathematically related to the ratio ($\left[{H}_{L}^{+}\right]/\left[{H}_{pB}^{+}\right]$) of TELP concentration $\left[{H}_{L}^{+}\right]$ at the liquid-membrane interface to the bulk-phase proton concentration $\left[{H}_{pB}^{+}\right]$ in the intermembrane space/crista space at the same *p*-side of the mitochondrial inner membrane, which is quite surprising.

When the TELP-associated thermotrophic activities utilize mitochondrial environmental heat energy (*k*_*B*_*T*) in driving the molecular turbine of F_0_F_1_-ATP synthase for the synthesis of ATP from ADP and Pi , as discussed also in the recent publication^13^ “a fraction of the environmental heat (*k*_*B*_*T*) energy may consequently be locked into the chemical form of energy in ATP molecules; and it would thus result in a small drop in the environmental temperature theoretically because of the TELP-associated isothermal environmental heat utilization”. However, in mitochondria and the cells, “there are many other processes (including the glycolysis, tricarboxylic acid cycle, and the redox-driven proton-pumping electron transport activities as well as the ATP utilization processes such as ATP hydrolysis) releasing heat energy, which could mask the thermotrophic function that features as the isothermal environmental heat energy utilization process”. Therefore, the energetic phenomenon in mitochondria (and the cells) may likely represent an interconnected mixture of both the chemotrophic processes and the thermotrophic processes. This subtle complexity could probably also explain why it took so long for the human beings on Earth to finally understand and figure it out here.

Based on the phosphorylation potential of + 15.6 kcal/mol (translating to − 65.3 kJ mol^−1^ ÷$F$  =  − 677 mV) previously used by Slater^81^ that is remarkably close to the magnitude of the critical free energy (− 63.5 kJ mol^−1^) for ATP hydrolysis in a functional animal heart cell obtained by Wu et al.^85^, the energy efficiency for the utilization of total pmf (including local pmf) in driving the synthesis of ATP can now be estimated. For example, according to the data in Table 2, at a mitochondrial membrane potential of 100 mV where the total pmf is 430 mV (including the local pmf of 324 mV), the energy efficiency for the utilization of total pmf in driving the synthesis of ATP is now estimated to be about 60% (677 × 100%/(2.67 × 430)), which thermodynamically appears to be a quite reasonable energy conversion efficiency. This also indicates that a substantial portion of the total pmf energy including the local pmf energy from TELP associated with the isothermal utilization of the mitochondrial environmental heat-related molecular motion kinetic energy (k_B_T) can indeed be locked to the ATP chemical energy.

Therefore, the discovery of thermotrophic feature isothermally utilizing environmental heat energy reported here “may have profound scientific and practical implications in bettering the fundamental understanding of bioenergetics and energy renewal” as recently pointed out for sustainable development on Earth^8^. With the new knowledge learned from this study, it may be possible to have the benefits of “mimicking this biophysical molecular scale process to create a new way in producing useful energy by isothermally utilizing the environmental heat energy from the ambient environment”^8,9,102^. It may also have scientific implications on the questions of who we are and how life began on Earth in terms of protonic bioenergetics. We now know that “water serves not only as a solvent and a substrate, but also as a protonic conductor” in living organisms including mitochondria^5,7^. The thermotrophic function isothermally utilizing environmental heat energy discovered herein was previously believed to be impossible for centuries. We now understand it is water as protonic conductor and consequently the formation of a protonic membrane capacitor with TELP that makes this thermotrophic function possible.

### Conclusion with the discovery

The classic Mitchellian chemiosmotic theory and its textbook pmf equation (Eq. 1) cannot fully explain the energetics even in mitochondria. The TELP theory^1,2,4,5,7,9,13^ with our updated pmf equations (Eqs. 2−5) can now fully elucidate the energetics of “oxidative phosphorylation in mitochondria” for the entire range of membrane potential ($\Delta\psi$) from 50 to 200 mV (Table 2 and Fig. 1). Through this study, we have now identified a novel thermotrophic function featured as “the isothermal utilization of environmental heat energy associated with the thermal motion kinetic energy of TELP in driving the synthesis of ATP” in mitochondria.

It is now, for the first time, numerically showed that the local pmf associated with TELP isothermally utilizing their environmental thermal motion kinetic energy represents about 75% of the total pmf which is the sum of the classic pmf and local pmf. The local pmf is now calculated, for the first time, to be in a range from 300 to 340 mV while the classic pmf is in a range from 60 to 210 mV in mitochondria with membrane potentials in a range from 50 to 200 mV, respectively. Apparently, a substantial fraction of the “environmental heat energy (*k*_*B*_*T*)” associated with TELP is “locked into the chemical form of energy in ATP molecules”.

Therefore, it is now quite clear that the low-grade environmental heat energy (i.e., the thermal motion kinetic energy of TELP) associated with the 37 °C human body temperature can be isothermally utilized by mitochondria “to perform useful work driving the synthesis of ATP”. As shown in an example with a transmembrane potential of 100 mV, mitochondria obtain as much as 324 mV (translating to the Gibbs free energy change of − 31 kJ/mol) of local pmf through isothermally utilizing environmental heat (protonic thermal motion kinetic energy), which surprisingly represents 75% of the total pmf (430 mV, translating to the Gibbs free energy change of − 41 kJ/mol); and only 25% is from the classic Mitchellian pmf component (106 mV, translating to the Gibbs free energy of − 10 kJ/mol). Consequently, we human as a mitochondria-powered organism is not only a chemotroph but also has a significant thermotrophic feature isothermally utilizing environmental heat energy in our human body environment to do work such as ATP synthesis.

Thermotropy of cells utilizing environmental heat energy was previously thought to be impossible for centuries. We now understand it is water as protonic conductor and thus the formation of protonic membrane capacitor that makes this thermotrophic feature possible. The energy efficiency for the utilization of total pmf in “driving the synthesis of ATP” is estimated to be about 60%, which again indicates a considerable fraction of the mitochondrial “environmental heat energy” associated with the thermal motion kinetics energy (*k*_*B*_*T*) of TELP being “locked into the chemical form of energy in ATP molecules”. Therefore, this discovery may have profound scientific implications.

Quite clearly, one of the immediate scientific implications from this discovery is that we human as a mitochondria-powered organism is not only a chemotroph, but now understandably also carries a significant thermotrophic feature!

## Data Availability

All data generated or analyzed during this study are included in this published article and its Supplementary Information (xls efile of excel spreadsheet showing how the protonic motive forces were calculated) available online at the journal website.
